# Association between electromagnetic field exposure and abortion in pregnant women living in Tehran

**Published:** 2016-05

**Authors:** Masoumeh Abad, Hossein Malekafzali, Masoumeh Simbar, Hassan Seyed Mosaavi, Effat Merghati Khoei

**Affiliations:** 1 *Department of * *Reproductive Health, School of Nursing and Midwifery, Shahid* *Beheshti University of Medical Sciences, Tehran, Iran.*; 2 *Department of * *Epidemiolog* *y * *and Biostatistics, * *School of Public Health, Tehran University of Medical Sciences, Tehran, Iran* *.*; 3 *Department of * *Reproductive Health, Shahid* *Beheshti University of Medical Sciences, Tehran, Iran.*; 4 *Department * *of Telecommunications engineering, Islamic * *Azad University, Garmsar* * Branch, Garmsar* *, Iran* *.*; 5 *Department of * *S* *exual * *H* *ealth * *P* *romotion, Iranian National Center for Addiction Studies (INCAS), Risk Behavior Reduction Institute, Tehran* *,* * Iran* *.*

**Keywords:** *Electromagnetic field*, *Pregnancy*, *Abortion*

## Abstract

**Background:**

Health-related quality of life is affected by electromagnetic field exposure in each person everyday life. However, this is extremely controversial issue.

**Objective:**

Investigation of the associations between electromagnetic field exposure and miscarriage among women of Tehran.

**Materials and Methods:**

In this longitudinal study, 462 pregnant women with gestational age <12 wks from seven main regions of Tehran city in Iran with similar social and cultural status were participated. Women were interviewed face-to face to collect data. Reproductive information was collected using medical file recorded in those hospitals the subjects had delivery. The measuring device measured electromagnetic waves, Narda safety test solutions with valid calibration date at the entrance door of their houses.

**Results:**

A significant likelihood of miscarriage in women who exposed to significant level of electromagnetic wave. However, this association was not confirmed by Wald test.

**Conclusion:**

This study may not provide strong or consistent evidence that electromagnetic field exposure is associated or cause miscarriage. This issue may be due to small sample size in this study.

## Introduction

It is about three decades that technology has introduced a modern event called cellphones to the modern life of human beings. According to the World Bank Group the pace of this developing technology has been so fast that the number of cellphone users has grown from one billion in 2000 to the number of 6 billion until now. About 5 billion out of this quantity is living in developing countries (1). 

Using this technology (telephone cell, Internet, radio, and television) requires integrated telecommunication antenna and therefore, the effects and outcomes of the waves produced by them is a hazard to human health. The vast increasing of satellite and earth telecommunication, broadcasting systems, cell phones, and other telecommunication systems have exposed houses, work places and promenades to radiations caused from electromagnetic signals (1). 

Electromagnetic fields have entered into human life more than acceptable limits. According to the powerful absorption rate and the noticeable spread of electromagnetic fields, extremely low frequency it is necessary to non-investigated their probable effects on biological systems more than ever. The effects of severe exposure to these fields have been indicated in some old books, but the effects of the long time exposure to the protected waves have been considered recently. Clinical trials in different parts of the world have shown that different physiologic processes like reproductive ability, fertility, fetus genesis process, and the balance of nervous and hormonal systems and some cardio respiratory factors can be affected inappropriately by these environmental factors and cause different anomalies directly or indirectly (2). 

Each kind of wavelength with high density can affect infertility. One of these kinds of waves is electromagnetic waves that are different on the base of density and length. The waves with short length are more harmful than the waves with high length. We live in a world full of radiations and more than 80% of them that threaten our lives are natural, but the effect of these radiations are different on vulnerable groups like children, old people and pregnant women (3). Telecommunication transmitters, radar systems and radio television transmitters, which have swing with high frequency, can make powerful magnetic fields, which are between 200 kHz to 10 GHz. The electromagnetic energy is absorbed by our bodies and is converted to thermal energy. If the absorption rate goes over 4 watt per kilogram, then the body temperature increases 1 0r 2 centigrade degree. So the waves between 27 MHz to 250 MHz can be used for treatment purposes.

Pregnant women and fetuses are both together vulnerable, because the radio frequency radiation (RF) is in interaction with cells of embryo’s development. Microwave radiation can damage to placenta barrier. The membrane prevents the transfer of substances between blood, and this fact mentions that a pregnant woman should not use cell phone except in case of emergency. According to recent findings, there is an association between cell phone use by mothers during pregnancy and greater chance of miscarriage, congenital anomalies, and behavioral problems in children. Spontaneous miscarriage (SM) is the most common complication of early pregnancy. Pregnancy loss before the 20th week of gestation is defined as spontaneous abortion, or miscarriage (4). The incidence of SM by the 20th of gestational weeks is 12 to 15 percent (5).

Risks related to SM are controversial. Some reported its association with the number of pregnancies. Other findings consider women at risk with more than 3 pregnancies, in contrast with those who reported the high incidence of SM in the first pregnancy (6%); however, low incidence of miscarriage has been reported among women with history of healthy pregnancy (6, 7). Overall, two of etiologic factors have been found out are uterine malformations and parental balanced chromosomal rearrangements (5).

Electromagnetic fields (EMF) exposure is alarmingly rising in the last decades. Current reports arguably suggest that environmental pollution so called electromagnetic waves can affects individual's health. Studies on both direct and indirect effects of EMF consisted of direct effects resulted from direct interaction of fields with the body, and indirect effects involved interactions with an object at a different electric potential from the body. Depending upon the frequency of the field, the physical quantities used to specify these restrictions are current density (J), specific energy absorption rate, and power density (S). Only power density in air, outside the body, can be readily measured in exposed individuals.

Reference levels: these levels are provided for practical exposure assessment purposes to determine whether the basic restrictions are likely to be exceeded. Some reference levels are derived from relevant basic restrictions using measurement and/or computational techniques, some address perception and adverse indirect effects of exposure to EMF. The derived quantities are electric field strength (E), magnetic field strength (H), magnetic flux density (B), power density (S), and currents flowing through the limbs (IL).

Far field-exposure is the main concern of those who believe in EMF's impacts on exposed individuals' health due to their lack of control on EMFs. Recently a review on "the association between radiofrequency electromagnetic field (RF-EMF) exposure and health-related quality of life" concluded that health-related quality of life is unlikely to be affected by radiofrequency electromagnetic field exposure in people's daily life (8).

The large population-based studies found no association between residential proximity to power transmission lines and video display terminals with the birth outcomes pre-term birth, Low Birth Weight (LBW),small for gestational age (SGA) birth or infant sex, spontaneous abortions or congenital anomalies) (9, 10). Some of the studies put emphasis on the impact of MFs on early pregnancy loss (11). Measurement of video display terminals MFs, however, indicated that when a woman was exposed to a video display terminals with a high MF level [>9 milligauss (mG)] during pregnancy, she had a more than 3-fold increased risk of miscarriage (12). 

In an Iranian case-control study with 58 matched women with unexplained spontaneous abortion at <14 wks, the researchers found significant difference between the women exposed to the magnitude of extremely low frequency electromagnetic fields in their houses and in the control group. They concluded a likelihood of association between extremely low frequency electromagnetic fields exposure and early spontaneous abortions (13). Another case-control study with 138 women in China indicated the increase risk of embryo growth ceasing significantly in exposing to EMFs (14).

The contentious epidemiologic findings on potential reproductive adverse effect associated with low-frequency electric and magnetic fields have been disseminated. Furthermore, studies about the association between EMFs exposure and the risk of SM are limited. Most of the researches on this field have been done on animal model and are not exactly extensible to human beings. Other researches also have done some studies about the effects of cell phones and other electromagnetic appliances on pregnancy outcomes. However, our research is the first cohort one in Iran that has investigated the association between waves made by broadcasting antennas and BTS and spontaneous abortion. 

On this basis, there is nor general agreement in this regard. Therefore, it’s a scientific necessity to continue further researches on this behalf, and evaluating the side effects of these radiations and its association with pregnancy outcomes.

## Materials and methods

We received Human Research Ethics Committee approval for this longitudinal study. We carried out the study with eligible women living in four main regions in Tehran, Iran. To fulfill inclusion criteria, study participants had to be 18-35 years of age; gestational age of 12 complete weeks or less, be Iranian and residents of Tehran, and interested in participating in the study. 

The participants were excluded from the study if they had been diagnosed with chronic diseases, any form of sexually transmitted infections, or became pregnant by artificial reproductive techniques due to infertility. Sample size was calculated on the base of Peduzzi reference according to this reference for the logistic regression model in which the effect of association of electromagnetic waves with response variable was considered (15). In this study, as the samples were almost the same in social and economic situation, so the effects of confounding variables were controlled. If we want to estimate three parameters in the model (spontaneous abortion, Intra Uterine Fetal Death, LBW), considering by a sample size we can estimate four parameters in L/R with α= 0.05 and 80% power. 

At the beginning of this study, 475 eligible women were identified for recruitment through seven medical centers located in four regions of Tehran. Written consent form was obtained from the subject if she was eligible to be screened. Women were screened for EMFs using NARDA at the entrance door of their houses three times during the pregnancy: semesters 1, 2, 3. An individual letter describing the purpose and procedure of the study was given to every woman who provided the researcher with her pregnancy test result. The first author to be screened for the eligibility contacted those women agreed to enter the study. 

Of these women, 413 of them completed an in-person interview and EMF measurement. In-person interviews were conducted by well-trained midwives to collect data on demographic details, identified risk factors for miscarriage, history of reproductive and medicals issues, and details about pregnancy in each of the trimesters. Throughout the study, 62 women due to unwillingness to cooperate, not recognizing the address (relocation), immigration, hypothyroidism, and husband’s opposition were excluded. At the end of the study, 413 women were included in the final analysis. The incident power density of the electromagnetic wave were measured using full calibrated tools NARDA SRM-3000 in safety evaluation mode, which is well known selective radiation meter in the range of 27MHz-3GHz and is approved by the International Commission on Non-Ionizing Radiation Protection (ICNIRP). 

The measurement of electromagnetic waves was done within the residential locations of the 413 samples, very close to the entrance door of their home, according to the standard instructions of ICNIRP (the allowed rate of exposure to RF radiation is 2 watts per kilogram for a 100 grams volume in 6 minutes (16). The environment was considered as the worst case and supposed that all the power Event-Related Potential (ERP) of all the antennas in a site have concentrated beginning of the study, the measurement was going to be done 3 times (one time in each trimester). 

However, after 2 times of measurement, as the results were all the same, at the third time the results were ignored and just one of the results was considered enough to be included in the study. Power density in Watts per centimeter square were measured for all of wireless services in the scope of this article very close to European Band Services, TV Band I, FM-Radio, Mid Wave, Paging, Band III (DVB-T), Trains, Band IV (DVB-T), Band V (DAB), GSM-R, GSM 900, L-Band (DAB), GSM 1800, DECT, UMTS-TDD, UMTS DL, W-LAN, ISM. Then, the measured values divided into three types of coupling mechanisms to the human body:

- Coupling of EMF wave energy divided into 4 Items such as: 1) Analogue Radio and Television Broadcast Services in central frequency of 100 MHz in W/cm2; 2) Digital Radio and Television Broadcast Services in central frequency of 650 MHz in W/cm2; 3) Mobile Communications Services (2G and 3G) in central frequency of 1.5 GHz in W/cm2; 4) Wi-Fi Access and MISC in central frequency of 2.45 GHz in W/cm2.
- Coupling of magnetic fields for frequencies less than 300 MHz in A/m.
- Coupling of electrical fields for frequencies less than 100 MHz for average body height of 160 cm in mA.

The total value for EMF wave power density resides in the range of 16 micro Watts per centimeter square, which is too much less than the ICNIRP limit line around 440 micro Watts per centimeter square. 

Pregnancy outcomes were included: spontaneous abortion, LBW, preterm delivery, and Intra Uterine Fetal Death .The pregnancy outcomes for all women were obtained through the following means: reviewing medical files at the hospitals where the participants gave birth to the child.

**Statistical analysis**

In univariate analysis, independent sample t-test was used for comparing the mean of numerical variable in women with and without abortion and Pearson Chi-square test was used for comparing the categorical variables between two groups. For multi variety, we conducted four sets of models in order to identify important demographic, pregnancy and wave factors predicting abortion. Abortion was considered as response variable in all models. We applied a logistic regression model for predicting the effective factors of abortion. Variables, which had P-value less than 0.20 in univariate analysis were entered into the multiple models. Four logistic regression models were fitted for the response variable. To avoid multi-colinearity and computational problems, standardized forms of wave variables were used in analysis. The followings are detailed descriptions of the four models:

- Model 1: This is a model of pregnant women with only the constant term in the model. Abortion was considered as response variable.
- Model 2: This is the same as Model 1 with the added variables related to the demographic and general women characteristics. These features were ages of women and familial relationship. So, this model assessed the effect of the woman-related variables on abortion.
- Model 3: This is the same as Model 2 with the added variables pertaining to the duration of pregnancy. Couple contention was added to the model at this stage and the effect of this factor was calculated adjusting for woman-related factors.
- Model 4: This is the same as Model 3, but with the added variable(s) for wave factors. In Model 4, it was added the total amount of waves into the multiple logistic regression models adjusting for women and pregnancy factors.
- Assuembly, missing data were missed completely at random and completed cases were used for the final analysis. The likelihood ratio and Wald tests was employed for fitness of the models.

## Results

The mean age of women was 28.22±4.53 years old. The frequency of spontaneous miscarriage was 56 cases. The incidence of abortion was 12.3%. Firstly, the risk of miscarriage associated with the radiation meter in the range of 27MHz-3GHz and the range of 16 micro Watts per centimeter square. Secondly, potential threshold effect of EMF exposure was examined to find the association of MF with the risk of miscarriage. At place, measurements did not indicate a consistent pattern of an association between increased exposure level and the rate of miscarriage. Univariate analyses revealed that wave factors were not significantly different in abortion and non-abortion group (p=0.42) (Table II). 

Multiple logistic regression models showed that the ages of pregnant women was a very significant factor in all models (p<0.001). Women, who had miscarriage, were significantly 55 months older than those without miscarriage (Table III). Familial relationship had a borderline significant effect on abortion in model 2 (18.8% vs. 11.1%, p=0.063) when pregnancy period factor was added in model 3 and wave factor in model 4, the effect of familial relationship became significant. The incidence of abortion in cases with familial relationship was found two times of those without familial relationship. Serious couple contention increase the risk of abortion in about 3 times (p=0.002).

In model 4, total wave services were added to the model in standardized form. Wald test showed no significant effect to total wave services on abortion (OR=1.12, p=0.447). However, when the maximum likelihood ratio test in logistic regression was applied a high significant decrease in 2 log likelihood observed (χ^2^LRT=6.96, df=1; p=0.008). Therefore, a controversy was seen in the results based on two different statistical analysis, Wald test and likelihood Ratio Test (LRT), which was probably related to the sample size.

**Table I:** Univariate analysis for quantitative and qualitative variables

**Variables**		**Miscarriage**		**p-value**
		**No (n=405)**	**Yes (n=57)**	
Diastolic blood pressure (mm hg)*		69.71 ± 8.68	66.11 ± 9.79	0.006
Systolic BP (mm hg)*		107.79 ± 9.98(	108.8 ± 9.36	0.487
Weight gain during pregnancy (Kg)*		1.66 ± 5.37	2.15 ± 5.50	0.539
Using complements during pregnancy**				
	Yes	317 ± 88.1	43 ± 11.9	0.409
	No	60 (84.5)	11 (15.5)	
Miscarriage symptoms during pregnancy**				
	Yes	43 (64.2)	24 (35.8)	**>**0.001
	No	334 (91.8)	30 (8.2)	
Sever vomiting**				
	Yes	62 (93.9)	4 (6.1)	0.086
	No	316 (86.3)	50 (13.7)	
Traveling during the pregnancy **				
	Yes	190 (91.8)	17 (8.2)	0.007
	No	182 (83.1)	37 (16.9)	
Smoking during the pregnancy **				
	Yes	7 (87.5)	1 (12.5)	0.370
	No	366 (87.4)	53 (12.6)	
Family quarrel during the pregnancy **				
	Yes	31 (72.1)	12 (279)	0.001
	No	342 (89.1)	42 (10.9)	
Any disease during the pregnancy **				
	Yes	54 (91.5)	5 (8.5)	0.198
	No	302 (95.6)	14 (4.4)	

* Data presented as mean±SD.

** Data presented as n (%)

**Table II:** Associations of EMFs and Miscarriage (T-test

**Electromagnetic fields**	**Miscarriage**		**p-value**
	**No (n=405)**	**Yes (n=57)**	
Coupling of low frequency magnetic fields	0.0001319291(0.0001304204)	0.0001319291(0.0000154454)	0.53
Coupling of low frequency electric and fields	0.5647905206(0.035880847)	0.5675585715(0.0269017396)	0.51
Service-sum	0.0000157265(0.0000006556)	0.0000157571(0.0000005694)	0.73
Analogue radio and television broadcast services in central frequency 100 MHz	0.0000071335(0.0000003262)	0.0000071396(0.0000002803)	0.89
Digital radio and television broadcast services in central frequency 650 MHz	0.0000031763(0.0000001379)	0.0000031798(0.0000001125)	0.85
Mobile communications services 1.5 GHz	0.0000034404(0.0000001722)	0.0000034509(0.0000002095)	0.67
Wi-Fi access and MISC in central frequency 2.45 GHz	0.0000019762(0.0000000932)	0.0000019866(0.0000000776)	0.42

* Data presented as mean(STD)

**Table III:** Result of logestic regression

**Variable**	**Model 1**		**Model 2**		**Model 3**		**Model 4**	
	**p-value**	**(OR)** **(95%) CI**	**p-value**	**(OR)** **(95%) CI**	**p-value**	**(OR)** **(95%) CI**	**p-value**	**(OR)** **(95%) CI**
Constant variable	<0.001	0.14	<0.001	0.003	<0.001	0.002	<0.001	0.001
Age (Year)	---	---	0.001	1.14 (1.05-1.23)	0.001	1.15 (1.06-1.24)	0.001	1.16 (1.07-1.26)
Gravid reference group (Not PP)	---	---	0.835	1.07 (0.58-1.98)	0.895	0.96 (0.51-1.81)	934	1.03 (0.54-1.94)
Familial relationship (Nil Ref Group)	---	---	0.063	1.93 (0.96-3.85)	0.050	2.03 (1.00-4.13)	0.023	2.31 (1.12-4.74)
Family conflict (Nil Ref Group)	---	---	---	---	0.002	3.39 (1.57-7.31)	0.001	3.93 (1.80-8.73)
*Total services (Wald test)	---	---	---	---	---	---	0.447	1.12 (0.83-1.52)
-2log likelihood	345.21		309.02		292.88		285.92	

* Amounts of waves standardized and entered the model.

## Discussion

The main objective of this paper was finding associations between EMF exposure and miscarriage. Recent findings suggest that women who exposed to very high-intensity EMFs have an increased risk of miscarriage. Early investigations put emphasize on the adverse effect of EMF on prenatal outcome, spontaneous abortion in particular. A population-based cohort study measured MF exposure with prospectively and indicated increased SAB risk associated with a MMF exposure level of 316mG (17). 

Li *et al* claimed that there is an association between miscarriage and EMF. They pointed out the maximum magnetic field and its effect on miscarriage rather than the effect of elevated spot EMF. They reported six-fold increased risk of spontaneous abortions among women exposed to magnetic fields of 16 mG or higher. However, any adverse effect of EMF with the threshold of 16mG or greater was shown in pregnancy less than 10 weeks, when emberyos' sensitivity is higher to its environmental factors (18).

The results of this study were compatible with a plausible link between EMF and miscarriage. However, previous research on exposures has focused on spontaneous abortions or congenital anomalies, and has not supported a significant association (19, 20) Similar to the present study, current investigations also reported on this association further more cautious. Few studies have examined the possible link between EMF exposure and miscarriage risk. The recent research did not indicate that health-related quality of life was affected by radiofrequency electromagnetic field exposure in people's everyday environments (21). Two reports in the 1980s found that using an electric blanket could increase the risk of miscarriage, but the results of more recent studies have been mixed (22). 

In a nested case-control study, (177 cases, 550 controls) conducted in the northern California Kaiser Permanente medical care system; it was indicated that wire codes and area measures association was weak. For the personal metrics (30 wks after last menstrual period), positive associations were observed (23). The national data records reported that out of 1.7 million pregnancies occurred in Iran yearly about 250000 are associated with miscarriages and illegal abortions compared with 12.3% found among our participants (24). 

A large population-based study showed that residential proximity to power transmission lines was not associated with an elevated likelihood of PTB, LBW, SGA birth, or infant sex (9). In sum, the current results suggested some associations between residential exposure to EMF and adverse birth outcomes, miscarriage as the common outcomes. It would not be claimed any cause and effect relationships between EMF and the subjects' miscarriages since some other traits (i.e. age of mother, familial marriage, and interpersonal conflicts) were significant. Future works need to be done examining the occupational exposure to EMF and other studies related to wireless devices and birth outcomes are suggested. Despite our findings, the evidence that EMFs increased the risk of miscarriage was uncertain.

The theories that empower the probable existence of association between EMF and abortion are:

1. The chemical interaction in cell membrane

2. Decrease of permeability in cell membrane and consequently decrease of cellular connections.

3. Increase in free radicals. 

4. Disorder in mitotic divisions

5. Damage to cellular proteins and cellular disconnection (21).

The other theory that is about the association of EMF and abortion is that the depth of penetration of the waves increases in tissues with less water.

The results, which were gained in our study, can be in accordance with one of the theories that have been mentioned above. These results matched with some researches, in which the association between EMF and abortion was obvious. In a similar study 900 pregnant women were studied with gestational age less than 10 weeks. all the exposed waves were measured by a special monitor and the result showed that these women were at 80% higher risk of abortion than other pregnant women. The increase of more than 16 MG in electromagnetic waves increased the risk of abortion, even more than the women with previous abortion or infertility history. Borbely, also, reported the increase of abortion in women who were exposed to video display terminals more than 4 hours (25). This study seems to differ with some other researches in this field due to sample size or challenges with the waves measuremnet.

## Conclusion

The findings of the present study would add to the body of knowledge in its own kind, as the recent systematic review claimed that "the effect of EMF on miscarriage/pregnancy loss has been studied only to a limited extent" (26). The electromagnetic waves had a significant association with the increase of abortion in women who were exposed to these waves (p<0.01). The likelihood test confirmed the effect of EMF on increasing chance of abortion, but we may need to have more comprehensive investigations on this field.

**Limitations**

Several potential limitations need to be considered at the time of the results interpretation of this study. First, the information on a woman's MF exposure was based on measurement at the subject's home door during the index pregnancy. Variation in MF level and daily activity pattern of women can be further limitations. The controversy in our study originates from not meeting some assumptions of statistical tests, such as normality of distribution, which is probably related to small sample size of statistics. 

## Note

***This article extracted from Ph.D. thesis. (Masoumeh Abad)***
